# Ghrelin-Derived Peptides: A Link between Appetite/Reward, GH Axis, and Psychiatric Disorders?

**DOI:** 10.3389/fendo.2014.00163

**Published:** 2014-10-27

**Authors:** Alexandra Labarthe, Oriane Fiquet, Rim Hassouna, Philippe Zizzari, Laurence Lanfumey, Nicolas Ramoz, Dominique Grouselle, Jacques Epelbaum, Virginie Tolle

**Affiliations:** ^1^UMR-S 894, Centre de Psychiatrie et Neurosciences, L’Institut national de la santé et de la recherche médicale, Université Paris Descartes, Paris, France

**Keywords:** ghrelin, eating disorders, food reward, alcohol and drug addiction, anxiety, depression, growth hormone

## Abstract

Psychiatric disorders are often associated with metabolic and hormonal alterations, including obesity, diabetes, metabolic syndrome as well as modifications in several biological rhythms including appetite, stress, sleep–wake cycles, and secretion of their corresponding endocrine regulators. Among the gastrointestinal hormones that regulate appetite and adapt the metabolism in response to nutritional, hedonic, and emotional dysfunctions, at the interface between endocrine, metabolic, and psychiatric disorders, ghrelin plays a unique role as the only one increasing appetite. The secretion of ghrelin is altered in several psychiatric disorders (anorexia, schizophrenia) as well as in metabolic disorders (obesity) and in animal models in response to emotional triggers (psychological stress …) but the relationship between these modifications and the physiopathology of psychiatric disorders remains unclear. Recently, a large literature showed that this key metabolic/endocrine regulator is involved in stress and reward-oriented behaviors and regulates anxiety and mood. In addition, preproghrelin is a complex prohormone but the roles of the other ghrelin-derived peptides, thought to act as functional ghrelin antagonists, are largely unknown. Altered ghrelin secretion and/or signaling in psychiatric diseases are thought to participate in altered appetite, hedonic response and reward. Whether this can contribute to the mechanism responsible for the development of the disease or can help to minimize some symptoms associated with these psychiatric disorders is discussed in the present review. We will thus describe (1) the biological actions of ghrelin and ghrelin-derived peptides on food and drugs reward, anxiety and depression, and the physiological consequences of ghrelin invalidation on these parameters, (2) how ghrelin and ghrelin-derived peptides are regulated in animal models of psychiatric diseases and in human psychiatric disorders in relation with the GH axis.

## Introduction

Psychiatric disorders are often associated with metabolic and hormonal alterations, including obesity, diabetes, metabolic syndrome as well as modifications in several biological rhythms including appetite, stress, sleep–wake cycles, and secretion of their corresponding endocrine regulators.

**Figure 1:**
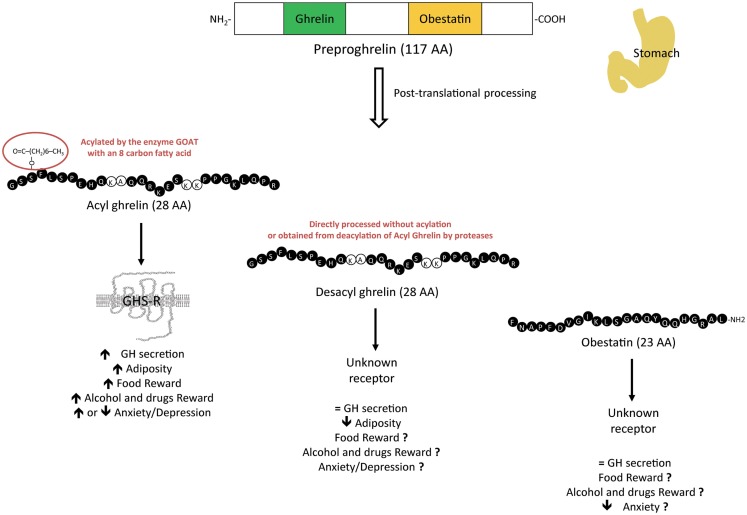
**Physiological effects of the different preproghrelin-derived peptides on GH secretion, adiposity, food reward, drugs reward, and anxiety/depression**. Preproghrelin is a unique prohormone as it encodes several preproghrelin-derived peptides with structural and functional heterogeneity. Whereas the contribution of acyl ghrelin, which binds to the GHS-R1a, has been well explored, the roles of the other variants, desacyl ghrelin, and obestatin, through receptors that still need to be identified, in these regulations need to be clarified.

At the interface between endocrine, metabolic, and psychiatric disorders, ghrelin plays a unique role as the only one increasing appetite. Ghrelin was initially identified as a gastrointestinal peptide originating from the stomach (1, 2) and as an endogenous ligand for the GH secretagogue receptor (GHS-R1a), the only ghrelin receptor identified so far. In addition to its primary effect as a GH secretagogue, ghrelin modulates many other neuroendocrine and metabolic functions: it is a powerful orexigenic and adipogenic peptide and a long-term regulator of energy homeostasis (3, 4). The actions of ghrelin on GH secretion and food intake require the addition of an eight-carbon fatty acid that is attached on a serine in position 3 by the enzyme ghrelin-*O*-acyltransferase (GOAT) (5, 6) and are exclusively mediated through the GHS-R1a. In addition, acyl ghrelin is processed from a 117 amino-acid prohormone that is unique in that it encodes other proghrelin-derived peptides with structural and functional heterogeneity. A naturally occurring molecule encoded by the same prohormone is desacyl ghrelin, which is the most abundant form in plasma (7) and initially thought to be an inactive peptide resulting from deacylation of ghrelin in tissues and blood (8). Another bioactive 23 amino-acid peptide is also derived from the same precursor and was originally proposed as the endogenous ligand for the GPR-39 (9). Although non-endocrine biological activities, such as regulation of glucose or lipid metabolism have been attributed to desacyl ghrelin or obestatin through receptors that still need to be characterized (10–12), some studies suggest that these derived peptides also antagonize the effects of acylated ghrelin on food intake and GH secretion (13, 14) (Figure 1).

The GHS-R1a is highly expressed in the hypothalamus, a region that controls neuroendocrine functions such as GH secretion, stress, and appetite, in the dorsal vagal complex that receives inputs from gut vagal afferents and also in the mesolimbic dopaminergic system of the ventral tegmental area (VTA) involved in addiction and reward (15, 16). Consistently, a recent literature shows that ghrelin is involved in stress and reward-oriented behaviors and regulates anxiety and mood. Thus, ghrelin is thought to adapt metabolism in response to emotional dysfunctions. Consistent with such hypothesis, the secretion of the gastrointestinal hormone and its derived peptides is altered in several psychiatric disorders (anorexia, schizophrenia) as well as in metabolic disorders (obesity) and in animal models in response to emotional triggers (such as psychological stress) but the relationship between these modifications and the physiopathology of psychiatric disorders remains unclear, especially since contradictory results arise from the literature. Interestingly, although the prevalence of most psychiatric disorders is not higher in obese individuals, the incidence of depression and anxiety is increased in this pathology (17).

The focus of this article is to review recent data concerning (1) the biological actions of ghrelin and ghrelin-derived peptides on food and drugs reward, anxiety, and depression by exploring pharmacological actions of the peptides in both rodents and humans and consequences of genetic or pharmacological blockade of the ghrelin/GHS-R system on these parameters, and (2) how ghrelin and ghrelin-derived peptides are regulated in human psychiatric disorders and animal models of psychiatric diseases such as eating disorders, anxio-depressive disorders, addiction to alcohol, and drugs of abuse. The link between ghrelin-derived peptides, GH axis, and psychiatric disorders will also be discussed.

## Preproghrelin-Derived Peptides in Eating Disorders

### Preproghrelin-derived peptides and food reward

Many psychiatric disorders are associated with an alteration in reward-related behaviors linked to the consumption of either natural reward (food) or drugs of abuse (alcohol, cocaine, nicotine, …). Both behaviors involve the meso-cortico-limbic system, including a dopaminergic projection from the VTA to the nucleus accumbens (NAcc).

#### Pharmacological actions of ghrelin in animals and humans

A significant number of studies suggest that ghrelin acts directly on the VTA to increase preference for and motivation to obtain highly palatable food but no data are available on the actions of the other ghrelin-derived peptides (Figure 1). In animal models, motivation to obtain rewarding food (like sucrose) is tested in an operant system by measuring lever pressing or nose poking in a progressive ratio paradigm. In such behavioral tests, either intraperitoneal (i.p), intracerebroventricular (i.c.v) or intra-VTA but not intra-NAcc ghrelin administration induces motivation for palatable food by increasing operant lever pressing for sucrose pellets or for 5% sucrose solution in rats (18–20). Similarly, reinforcement in satiated rats and motivation for highly palatable food even in food-restricted animals are increased after intra-VTA chronic ghrelin infusion (21). Interestingly, this operant response to sucrose pellets following acute intra-VTA ghrelin is prevented by forebrain dopamine depletion (6-OHDA) (22). In addition, ghrelin-induced increases in lever presses for a 5% sucrose solution is inhibited by pre-treatment with the D1-R antagonist, SCH-23390, but not D2-R antagonist, indicating that ghrelin-induced motivation for food is mediated via D1-R dependent mechanism. However, the significant increase for food motivation/reward behavior observed after ghrelin injection in the VTA is abolished by a pre-treatment with D1- or D2-receptor antagonist, injected in the NAcc, a main target of VTA dopaminergic neurons (23).

Ghrelin can also modify food preference, that can be measured in rodents by a free choice feeding paradigm, where animals are given a free choice of either chow or highly palatable food. Indeed, acute bilateral intra-VTA injection of ghrelin in rats increases consumption of rewarding food (peanut butter) but not standard chow and i.c.v ghrelin-induced motivation for this rewarding food is reduced in VTA-lesioned animals (24). Furthermore, in a two-bottle open access paradigm, i.c.v ghrelin injections in rats increases consumption of a 5% sucrose solution and this is prevented by i.p administration of 18-methoxycoronaridine, a selective antagonist of α3β4 nicotinic receptors, known to reduce operant response to sucrose (25). However, whether the palatability or the energetic value of energy-rich food is increased by ghrelin is still unresolved. In a single bottle test, mice injected i.p with ghrelin show a significant increase in saccharin intake, in the presence or in the absence of regular food. In addition, in a free-choice preference test, preference for the non-nutritive saccharin-flavored jelly is enhanced by i.p ghrelin injection in mice, demonstrating that ghrelin can augment the overconsumption of a sweet palatable non-nutritive solution, even when presented without any source of calories (26). However, when rats are given the choice between a palatable yet low-calorie sucrose solution or a calorically dense chow, i.c.v or intra-PVN ghrelin administration results in an increased intake of chow but not sucrose, suggesting that the primary effect of ghrelin is to stimulate food to satisfy energy needs (27).

Hedonic and rewarding effects of ghrelin are also observed in humans. Indeed, ghrelin administered intravenously to healthy volunteers during fMRI increases the neural response to high-energy- and low-energy-food pictures evaluation task in brain regions involved in reward processing and hedonic feeding, including amygdala, orbitofrontal cortex, anterior insula, striatum and/or hippocampus (28, 29), and ghrelin effects in the amygdala are correlated with self-rated hunger score (28).

#### Genetic or pharmacological blockade of the ghrelin/GHS-R pathway

Suppressed intake of rewarding food in a free-choice food paradigm, lack of cue-potentiated feeding and suppressed motivation for food in an operant responding model in *ghsr*^−/−^ mice support a role of the endogenous peptide in hedonic eating [for review, see Ref. (30)]. Attenuated motivation for food in an operant responding model and decreased hedonic feeding response for a palatable high-fat dessert has also been described in mice invalidated for GOAT, the enzyme that acylates ghrelin, suggesting that a specific role for acyl ghrelin in this response (31).

### Regulation of preproghrelin-derived peptides in animal and human pathophysiology

#### Animal pathophysiology

In obesity, changes in food intake and reward-associated behaviors can be observed, often accompanied by alterations in eating patterns and increased intake of foods with high fat and sugar content [for review, see Ref. (32)]. Studies in animal models evidenced a dysregulation of the ghrelinergic pathway in obesity. Indeed, the long-term exposure to high-fat diet in the diet-induced obese mouse model leads to lower plasma acyl ghrelin (AG) and total ghrelin (TG) levels (Table 1), reduced hypothalamic GHS-R1a expression and suppressed feeding response to ghrelin injections (33, 34), indicating central ghrelin resistance. Interestingly, the modulatory action of ghrelin on reward on a progressive ratio paradigm is blunted in C57BL/6 mice with diet-induced obesity (35), supporting a dysfunction of the reward system and ghrelin resistance at the level of the reward circuit as well in obesity.

**Table 1:** **Regulation of plasma ghrelin concentrations in different mouse models, nutritional status, and experimental conditions**.

Type of study	Reference	Sex	Animal model or strain	Experimental conditions/stimulus	Nutritional status	Ghrelin secretion
Eating disorders/ obesity	(33)	M	Diet-induced obese C57BL/6 mice	12 weeks on HFD	NA	↘ AG and TG in DIO mice Hypothalamic ghrelin resistance
	
	(34)	M	Diet-induced obese C57BL/6 mice	12 weeks on HFD response to fasting	20 h Fasting	↗ AG in response to fasting in control and DIO mice
	
	(35)	M	Diet-induced obese C57BL/6 mice	13 weeks on HFD response to sucrose reward	Free access to food	Resistance to ghrelin-induced sucrose reward

Alcohol addiction	(36)	M	Wistar, Wistar high preferring (WHP), Wistar low preferring (WLP)	Naïve (no alcohol)	12 h fasting	↘ AG and TG in WHP compared to WLP and Wistar ethanol-naïve rats
	
	(36)	M	Wistar, Wistar low preferring (WLP), Wistar high preferring (WHP) ethanol naive rat	Acute ip ethanol injection	Free access to food	↘ AG and TG in Wistar and WLP rats
	
	(36)	M	Wistar alcohol-preferring (PR), Wistar non-preferring (NP), Wistar high preferring (WHP), Wistar low preferring (WLP)	Chronic alcohol consumption	12 h Fasting	↘ ↘ AG and TG in PR and WHP rats ↘ AG and TG in NP and WLP rats
	
	(37)	M	Voluntary chronic alcohol consumption in high-alcohol (alko, alcohol: AA) and low-alcohol (alko, non-alcohol: ANA) consuming rats	Continuous then limited access to increasing alcohol concentrations in a two-bottle-choice drinking paradigm for 14 weeks	Free access to food	↘ TG in AA rats ↘ ↘ TG in ANA rats
	
	(38)	M	Voluntary chronic alcohol consumption in Wistar rats	Access to 20% alcohol in a two-bottle-choice drinking paradigm during 10 months	Free access to food	Plasma ghrelin not assayed Negative correlation between GHS-R expression in the VTA and alcohol intake

Drug addiction	(39)	M	Drug self-administration in Lister hooded rats	Trained to self-administer cocaine iv	Restricted diet regime	Positive correlation between plasma ghrelin and cocaine-seeking behavior

Stress/anxiety/depression	(40)	NA	Caloric restriction	60% Caloric restriction during 10 days	60% Caloric restriction	↗ GA
	
	(40)	M	Chronic social defeat stress (CSDS)	10 days of CSDS	Free access to food	↗ GA associated with increased caloric intake and weight gain
	
	(41)	M	Chronic social defeat stress (CSDS)	10 days of CSDS	Free access to food	↗ GA and = GNA in both WT and KO Attenuated weight gain and feeding in GHS-R compared to WT
	
	(42)	M	High-anxiety Wistar Kyoto and low-anxiety SD rats	Unstressed	Free access to food	↘ TG in high-anxiety compared to low-anxiety rats
	
	(42)	F	High-anxiety Wistar Kyoto (WKY) and low-anxiety SD (SPD) rats	Acute exposure to water avoidance	Free access to food	↗ TG in WKY ↗ ↗ TG in SPD

*M, male; F, female; NA, not available; TG, total ghrelin; AG, acyl ghrelin; Ghrelin, no information on isoform measured*.

#### Human pathophysiology

**Figure 2:**
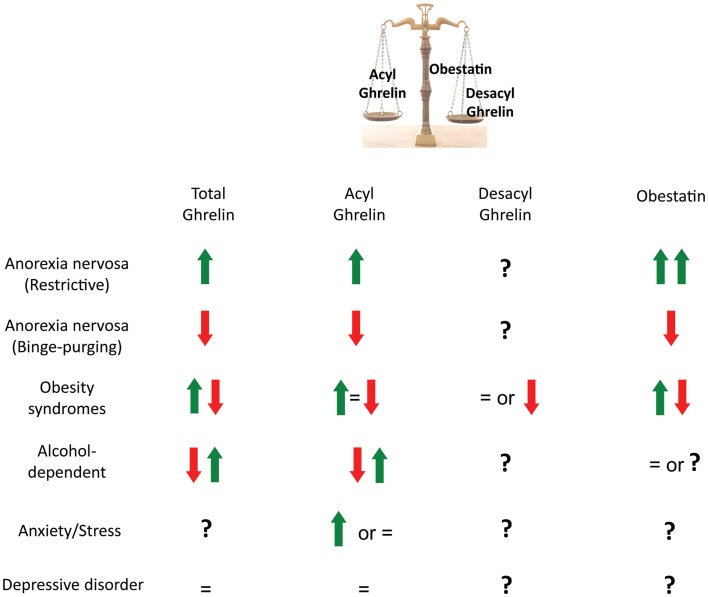
**Plasma levels of preproghrelin-derived peptides in metabolic and psychiatric disorders**. In anorexic patients, total ghrelin, acyl ghrelin, and obestatin are increased in the restrictive type and reduced in binge-purging. This may represent different abilities to adapt to starvation. In obesity syndromes, total ghrelin, acyl ghrelin and obestatin are either found reduced, unchanged, or increased. Discrepancies may be due to the variety of obesity syndromes (monogenic obesity, nutritionally induced obesity, presence of a metabolic syndrome, multifactorial disorders such as the Prader-Willi syndrome). In alcohol-dependent patients, ghrelin is found either reduced or increased compared to healthy subjects but there might be differences in gender, body mass index, nutritional/metabolic status and/or time after alcohol withdrawal from one study to another that can explain the contradictory results. In major depressive disorders, the majority of studies report no differences in plasma total or acyl ghrelin levels compared with healthy controls. Whether ghrelin can contribute to the degree of food craving, alcohol craving or depression is not clearly demonstrated as treatments and metabolic modifications can interfere with the results.

Dysregulation of the ghrelinergic system is also observed in human obesity [for review, see Ref. (43)] in relation with the alteration in the reward system (44) (Table 2). In most obese syndromes, low plasma ghrelin levels is due to a reduction in the desacyl form of ghrelin whereas acyl ghrelin is either increased, decreased or unchanged (45–47) (Figure 2). In contrast, in Prader–Willi syndrome, a rare genetic disorder characterized by multiple symptoms including severe binge-eating, growth retardation as well as learning disabilities, anxiety and depression, hyperghrelinemia correlates positively with hyperphagia (48, 49) and is mostly due to an increase in acyl ghrelin levels whereas desacyl ghrelin and obestatin are unchanged (50, 51). Although the origin of these elevated ghrelin levels remain unclear, it could result from undernutrition due to a failure to thrive during infancy.

**Table 2:** **Regulation of plasma ghrelin/obestatin concentrations in different human pathologies, health and nutritional status, and experimental conditions**.

Type of study	Reference	Sex	Subjects and health status	Experimental conditions/stimulus	Nutritional status/time of sampling	Ghrelin/obestatin secretion
Eating disorders/obesity	(52)	32 F	10 Obese (OB) 11 Anorexia nervosa (AN) 11 Healthy controls (HC)	Basal conditions	Morning after overnight fast	↘ G, ↘ AG, ↘ obestatin (OB) ↗ AG, ↘ DAG, ↗ obestatin (AN)
	
	(53)	41 F	10 Constitutional thinness (CT) 15 AN-R 7 AN-R recovered (PRAN) 9 HC	Basal conditions	Morning after overnight fast Circadian pattern (every 4 h)	↗ AG and TG, ↗ obestatin (AN) = AG, = obestatin (CT)
	
	(54)	57 F	22 AN-R 10 AN-BP 16 AN-BN 9 HC	Basal conditions	Morning after overnight fast Circadian pattern	↗ AG and TG, ↗ obestatin (AN-R) ↘ AG and TG, ↘ obestatin (AN-BP) ↘ AG and TG, ↘ obestatin (BN)
	
	(55)	25 F	9 AN, 6 AN recovered, 10 CT	Basal conditions	Morning after overnight fast	↗ TG (AN)
	
	(55)	25 F	9 AN 6 AN recovered 10 CT	Ghrelin infusion (5 pmol/kg × min) during 300 min	Morning after overnight fast	↘ GH response to ghrelin = Feeding response to ghrelin
	
	(56)	16 F	9 AN-R 7 HC	Basal conditions	Morning after overnight fast	↗ TG (AN)
	
	(56)	16 F	9 AN-R 7 HC	Acute ghrelin administration (1.0 μg/kg)	Morning after overnight fast	↘ GH response to ghrelin = Glucose response to ghrelin
	
	(57)	5 F	5 AN-R	Ghrelin infusion for 24 days: 3 μg/kg for 5 min during 14 days before breakfast and dinner	Morning after overnight fasting	↗ Hunger sensation evaluated as VAS score
	
	(45)	M F	Normal weight Obese, no metabolic syndrome (no-MS) Obese, metabolic syndrome (MS)	Basal conditions	Morning after overnight fast	↘ TG and DAG, = AG (MS) ↘ TG and ↗ AG (non-MS)
	
	(46)	34 M	Normal weight (17 M) Overweight (17 M)	Basal conditions	Fasting	↘ TG and DAG (in OW) = AG (in OW)
	
	(47)	101 M 79 F	Normal weight (31 M, 34 F) Obese, no metabolic syndrome (no-MS) (40 M, 20 F) Obese, metabolic syndrome (MS) (30 M, 25 F)	Basal conditions	Overnight fasting	↗ AG and ↘ DAG (non-MS) ↗ ↗ AG and ↘ ↘ DAG (MS)
	
	(48)	21 M 27 F	Prader–Willi syndrome (PWS) (10 M, 8 F) Obese (4 M, 10 F) Lean (7 M, 9 F)	Basal conditions	Overnight fasting	↗ TG (in PWS)
	(49)	M F	Prader–Willi syndrome (PWS) obese controls	Basal conditions	Fasting	↗ TG (in PWS)
	
	(50)	21	Prader–Willi syndrome (PWS) (11) Obese control children (10) Identical BMI	Basal conditions	Fasting	↗ AG = DAG
	
	(51)		Prader–Willi syndrome (PWS) (15) Obese control children (18) Identical BMI	Basal conditions	NA	↗ AG and TG = Obestatin

Alcohol addiction	(58)	4 M 4 F	Healthy subjects Non-obese (moderate social drinkers)	Acute oral (ethanol versus drinking water)	Morning	↘ TG
	
	(59)	6 M 6 F	Healthy subjects Normal BMI (moderate social drinkers)	Acute oral (ethanol versus drinking water)	Overnight fasting before and after ethanol	↘ AG and TG
	
	(60)	9 M	Healthy subjects Normal BMI	Acute oral (ethanol versus non-ethanol drink) + stress exposure	Early afternoon before and after ethanol	↘ TG
	
	(61)	5 M 5 F	Healthy subjects Non-obese (moderate social drinkers)	Acute oral (ethanol versus drinking water)	Morning fed before and after ethanol	↘ TG = Obestatin
	
	(62)	22 M 22 F	Healthy subjects Normal BMI (moderate social drinkers)	Acute intravenous (ethanol versus saline)	Morning fed	↘ TG, = obestatin No gender effect
	
	(63)	20 M	Healthy subjects: lean (11) or overweight (9)	Moderate alcohol during 3 weeks	Overnight fasting	↗ Ghrelin
	
	(64, 65)	142	Healthy control (24) Alcohol-dependent (early abstainers, 21) Alcohol-dependent (active drinkers, 97) Normal BMI	Chronic alcoholism	Overnight fasting	↗ Ghrelin in alcoholic compared to HC ↗ Ghrelin in early abstainers compared to active drinkers
	
	(66)	44 (M + F)	Healthy control (20) versus alcohol-dependent (24) Non-obese	Chronic alcoholism	Overnight fasting	↘ Ghrelin
	
	(67)	30 M	Healthy control (15 M) versus alcohol-dependent (15 M) Non-obese	Chronic alcoholism	Overnight fasting	↘ Ghrelin Positive correlation between ghrelin levels and alcohol craving
	
	(68)	115 M 39 F	Healthy control (12 F + 33 M) versus alcohol-dependent (27 F + 82 H) Normal BMI	Chronic alcoholism	NA	↗ Ghrelin (females) = Ghrelin (males) Positive association between ghrelin and alcohol craving
	(69)	97 M	Healthy control (50 M) versus alcohol-dependent abstainers (47 M) Normal BMI	>30 days of abstinence	Overnight fasting	↗ Ghrelin Positive correlation between ghrelin and duration of abstinence
	
	(70)	111 M	Healthy control (50 M) versus alcohol-dependent abstainers (61 M)	14 days of abstinence	Overnight fasting	↗ AG and = TG Positive association between AG and alcohol craving
	
	(71)	64 M	Alcohol-dependent abstainers (64 M) classified in normal glucose tolerance (NGT), pre-diabetes (pre-DM) and diabetes (DM) Normal BMI	>30 days of abstinence with rehabilitation treatment	Overnight fasting	↗ Ghrelin in NGT ↗ ↗ Ghrelin in pre-DM and DM

Drug addiction	(72)	11	Heavy smokers	Acute 24 h nicotine withdrawal	Fed a light meal 2 h before	No association between TG and craving or withdrawal symptoms
	
	(73)	123 M 143 F	Young adults Normal BMI	Intrauterine exposure to prenatal smoke	Smoking and food *ad libitum*	↗ TG
	
	(74)	54 M	Smokers (31 M) and non-smokers (23 M) Slightly overweight	Chronic smoking	Overnight fasting and abstinence from smoking	= TG
	
	(74)	54 M	Smokers (31 M) and non-smokers (23 M) Slightly overweight	Acute smoking (2 cigarettes)	Overnight fasting and abstinence from smoking	= TG (smokers) ↘ TG (non-smokers)
	
	(75)	24 M 26 F	Healthy non-smokers Normal BMI	Nicotine administration	Overnight fasting	= DAG

	(60)	9 M	Healthy subjects	Psychological stress = public speaking stressor	NA	= TG
Stress/anxiety/depression	(76)	8 M 16 F	Normal weight (8) Obese (8) Binge-eating (8)	Psychological stress = standardized trial social stress test (TSST) = public speaking	Morning after light breakfast	= TG after psychological stress Positive correlation between the change in TG and the change in cortisol
	
	(77)	103 F	Healthy subjects	Stress = public speaking	Food provided	↘ AG in emotional eaters ↗ AG in women anticipating the stressor compared to those not subjected to the stressor ↘ AG in non-emotional eaters following food consumption = AG in non-emotional eaters following food consumption
	
	(78)	68 M 61 F	Major-depressive disorder (MDD) (44 M, 39 F) Healthy subjects (24 M, 22 F) MDD slightly overweight	Basal conditions	Overnight fast	= TG in MDD Positive correlation between ghrelin and eating behavior scales TFEQ
Stress/anxiety/depression	(79)	18 M 22 F	MDD patients (9 M, 11 F) Healthy subjects (9 M, 11 F) Normal BMI	Basal conditions	Fed Overnight sampling	= TG in MDD
	
	(80)	M + F	MDD patients (9 M, 6 F) Healthy subjects (16)	Basal conditions		= AG or TG in MDD
	
	(81)	48 M + F	MDD patients (24) Healthy subjects (24)	Basal conditions	Overnight fasting	= AG in MDD Positive correlation between AG and the severity of reduced appetite in MMD
	
	(82)	64 F	Anorexic patients (15) Normal weight (32) Overweight (17)	Basal conditions	Overnight fasting	No relationship between TG and symptoms of depression or anxiety
	
	(83)	245 M + F	MDD or panic disorders Treatment responders (89) Treatment non-responders (59) Healthy subjects (97)	Basal conditions	Early afternoon No eating 60 min before	↗ AG in treatment-resistant patients
	
	(84)	12 M 12 F	MDD patients (24) Healthy subjects (22)	Basal conditions citalopram treatment (3 months)	Overnight fasting	↘ AG and TG in MDD ↘ AG and TG after treatment
	
	(85)	40 M	MDD lean patients (40 M)	Basal conditions maprotiline treatment (30 days)	Overnight fasting	↗ TG and weight gain after treatment
	
	(86)	M + F	Major-depressive episode (MDE, 16) Bipolar disorder manic episode (BD-me, 12) Healthy subjects (25)	Electroconvulsive therapy (ECT)	Overnight fasting	↘ AG after treatment in all subgroups but BMI unchanged

*M, male; F, female; BMI, body mass index; OB, obese; AN-R, anorexia nervosa restrictive-type; AN-BN, anorexia nervosa with episodes of bulimia; AN-BP, anorexia nervosa with episodes of binge-purging; HC, healthy control; CT, constitutional thinness; MDD, major-depressive disorder; NA, not available; AG, acyl ghrelin; DAG, desacyl ghrelin; TG, total ghrelin; Ghrelin, either TG or AG (?)*.

Anorexia nervosa is a major cause of undernutrition in young women. In most of the studies, plasma acyl ghrelin and obestatin levels are elevated in anorexic patients with a pure restrictive-type (AN-R) compared to control or obese subjects (52–54, 87) and an inverse correlation between acyl ghrelin or obestatin and body mass index (BMI) is found (52). In contrast to AN-R, in patients with binge-purging (AN-BP), acyl ghrelin is increased and obestatin levels reduced or unchanged (53, 54), suggesting different abilities to adapt to starvation in AN-R and AN-BP despite similar BMI (Table 2). Inability of ghrelin to induce appetite during intravenous infusion may suggest some resistance to the orexigenic peptide in AN-R patients (55). However, pharmacological effects of ghrelin in AN have not been conclusive yet as some studies also reported increased hunger sensations after ghrelin treatment (56, 57). Data should be interpreted with caution due to the small number of patients and the difficulty to evaluate the degree of hunger in this pathology. Other factors that may counterbalance hyperghrelinemia should be taken into account. Interestingly, it has been hypothesized that higher obestatin levels in the restrictive type may also contribute to the reduced hunger and/or reduced motivation to eat in this pathology (53, 54) (Figure 2).

Alterations in the serotonergic/dopaminergic signaling are described in AN patients (88, 89) and avoidance for food is positively correlated with the increased striatal D2/D3 receptor binding. The altered impact of ghrelin on the reward system could modify the integration of information related to emotional processes, as suggested by connections between the prefrontal cortex and the NAcc. Interestingly, it has been demonstrated that the heterodimeric formation of ghrelin receptor and dopamine D2-receptor is required for the anorexigenic effects of dopamine in hypothalamic neurons (90). The co-expression of these receptors in the mesolimbic dopaminergic system may also be important to modulate motivation and reward.

## Preproghrelin-Derived Peptides in Alcohol Addiction

### Preproghrelin-derived peptides and alcohol consumption

Several reviews already focused on the role of the ghrelin/GHS-R signaling in the rewarding properties of alcohol in animal models or humans (91, 92). A large number of studies demonstrate that ghrelin is involved in alcohol intake, showing altered plasma ghrelin levels in alcoholic patients or high-alcohol consuming rat strains as well as reduced alcohol intake in animal with disrupted ghrelin signaling. However, very little pharmacological data exist so far.

#### Pharmacological actions of ghrelin in animals and humans

Ghrelin, delivered either peripherally, centrally or directly in specific brain nuclei, increases reward-relevant behaviors such as alcohol consumption (92) (Figure 1). Central ghrelin administrations, i.c.v or directly in the VTA or laterodorsal tegmental area (LDTg), increase alcohol intake in a two-bottle (alcohol/water) free-choice paradigm where access to alcohol was limited to 90 min/day for 2 weeks in C57BL/6 mice, a strain with high-alcohol preference (93). Pre-clinical models termed “drinking in the dark” (DID) are developed to examine binge-like ethanol consumption in rodent populations (94). In this procedure where animals have a 2 h access to a 20% ethanol solution during the beginning of the dark period, neither food deprivation nor i.p administration of ghrelin altered drinking in C57BL/6J mice (95). In humans, intravenous ghrelin administration increases alcohol craving in alcohol-dependent heavy drinkers (96).

#### Genetic or pharmacological blockade of the ghrelin/GHS-R pathway

Genetic or pharmacological blockade of the GHS-R1a also reveals the importance of ghrelin signaling in alcohol-related behaviors. Pharmacological blockade with GHS-R1a antagonists reduces voluntary alcohol consumption and preference and suppresses reward induced by alcohol in both rats and mice [reviewed in Ref. (91)]. For example, JMV2959, a selective GHS-R1a antagonist, impacts both acute and chronic alcohol consumption. Acute injections of the antagonist reduce the operant self-administration of alcohol in rats, decreases high-alcohol consumption in two strains of alcohol-preferring rats, Long–Evans and alko alcohol (AA) preferring rats (97) as well as alcohol consumption after several months of exposure to alcohol (38). Chronic administrations of the antagonist decrease alcohol intake without inducing tolerance or rebound (38). In mice, in a two-bottle choice paradigm, the non-selective antagonist [d-Lys3]-GHRP-6 reduces preference to alcohol (98). In addition, GHS-R1a blockade with JMV2959 either peripherally or centrally reduces voluntary alcohol consumption and preference, alcohol-induced locomotor stimulation, accumbal DA release, and conditioned-place preference (CPP) (93, 99). Similarly, when compared to wild-type mice, *ghrelin*^−/−^ mice display lower ethanol-induced CPP and locomotor stimulation and reduced voluntary alcohol consumption and preference in a two-bottle choice test (99). The ability of alcohol to increase NAcc dopamine release is absent in *ghrelin*^−/−^ mice (100). The rewarding properties of alcohol are also reduced in the *ghsr*^−/−^ mice (93). However, Spiegelmer-neutralization of circulating ghrelin, thereby preventing its access to the brain, does not attenuate alcohol-induced locomotor activity, NAcc DA release, and CPP in mice, neither modifies alcohol consumption in a two-bottle free-choice paradigm in rats, suggesting that central rather than peripheral ghrelin signaling is preferentially involved in alcohol consumption (101). In contrast, Roux-en-Y gastric bypass (RYGB), which reduces circulating ghrelin levels, decreases ethanol intake and the reinforcing properties of ethanol in ethanol-preferring rats. In this model, pharmacological replacement of ghrelin restores drinking behavior (31), suggesting that endogenous circulating ghrelin is important in alcohol preference. It should also be noted that, in RYGB rats, a GHS-R1a antagonist, [d-Lys3]-GHRP-6, reduces operant performance to earn alcohol reward and alcohol consumption, suggesting that increased ghrelin might contribute to increased alcohol reward in such animals (102). Altogether, these data suggest that selective blockade of the ghrelin/GHS-R pathway could be a potential treatment for pathological alcohol consumption.

### Regulation of preproghrelin-derived peptides in animal and human pathophysiology

Although ghrelin induces addictive behaviors, including alcohol consumption in both rodents and humans, an important question is whether hyperghrelinemia is associated with addictive behaviors, such as alcohol drinking. Conflicting data concerning the involvement of ghrelin in the physiopathology of alcohol dependence have been reported (103, 104).

#### Animal pathophysiology

In rodents, chronic alcohol consumption leads to reduced total and acylated ghrelin levels in rats of different strains (PR: Wistar alcohol preferring, NP: Wistar non-preferring, and WHP: Wistar high preferring) but the lowest ghrelin levels are observed in PR and WHP strains (Table 1). Furthermore, there is an inverse relationship between ghrelin levels and alcohol intake (36). In high-alcohol (AA) consuming rats, reduction in plasma total ghrelin levels after alcohol exposure during several weeks is of lower amplitude as compared to low-alcohol (ANA) consuming rats (37). Interestingly, GHS-R1a expression in NAcc, VTA, amygdala, prefrontal cortex, and hippocampus is higher in AA rats, suggesting that the ghrelin pathway may be involved in alcohol susceptibility. But, after 10 months of voluntary alcohol consumption induced by intermittent access to alcohol in a two-bottle choice drinking paradigm, GHS-R expression in the VTA is significantly down-regulated in high- compared to low-alcohol consuming Wistar rats and a negative correlation is observed between GHS-R expression in the VTA and alcohol intake (38).

#### Human pathophysiology

**Figure 3:**
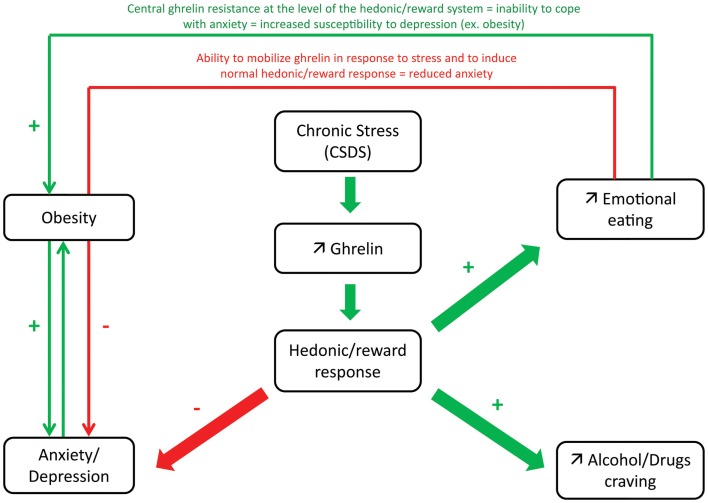
**Proposed model of hedonic/reward response to ghrelin after chronic stress in relation with anxio-depressive symptoms**. During chronic stress, increased ghrelin secretion has been shown to induce emotional eating by acting at the level of the hedonic/reward system. As ghrelin has been shown to have anxiolytic actions in response to stress, this adaptative response may contribute to control excessive anxiety and prevent depression. In obesity, lower ability to mobilize ghrelin in response to stress or central ghrelin resistance at the level of the hedonic/reward system may explain the inability to cope with anxiety and increased susceptibility to depression. Reciprocally, depressed subjects have increased susceptibility to obesity or eating disorders (due to altered hedonic/reward response). Elevated ghrelin may also contribute to alcohol/drug craving: higher ghrelin levels correlate with higher measurements of alcohol craving.

In healthy humans, acute alcohol consumption induces significant declines in total and acylated ghrelin concentrations as early as 15 min following alcohol ingestion (58–61), whereas obestatin levels are unchanged (61). Interestingly, fasting-induced increase in ghrelin levels is also reduced after intravenous alcohol administration in both males and females (62). In contrast, after moderate alcohol consumption during 3 weeks in healthy men, ghrelin concentrations are increased but both lean and overweight subjects were included in the analyses and plasma ghrelin levels tended to be lower in overweight associated with reduced insulin sensitivity (63). Data concerning the effect of chronic alcohol intake in alcohol-dependent subjects are conflicting. Indeed, alcohol dependence is associated with either increases or decreases in plasma ghrelin levels compared to healthy control subjects (65–67) (Figure 2) but higher ghrelin levels correlate with higher measurements of alcohol craving using the obsessive compulsive drinking scale (OCDS) (67, 105, 106) (Figure 3). Some difficulties in comparing data from the literature are that explorations are sometimes performed in different genders, various BMI, early abstainers versus active drinkers and at different times after alcohol withdrawal. The small number of subjects in some studies is also a factor to take into account (Table 2). Interestingly, a study comparing both genders found that ghrelin levels are higher in female alcohol-dependent patients only when compared to appropriate gender controls while no differences are found in males (68), suggesting that ghrelin response may be gender dependent. In addition, among alcohol-dependent subjects, ghrelin levels are higher in early abstainers compared to active drinkers (65). Although most hormonal explorations are performed after an overnight fast, nutritional status at the time of sampling may also contribute to differences observed. Finally, differences in ghrelin secretion observed in alcohol-dependent patients from one study to another may also be a consequence of the loss of metabolic control as alcohol intake is compensated by a decrease in non-alcoholic nutrient intake (66). Consumption of other substances of abuse can also interfere with plasma ghrelin levels and although smoking is an exclusion factor in some studies (62), others have to take into account the smoking status of the participants (70).

Whereas alcohol intake seems to reduce plasma ghrelin levels, alcohol withdrawal induces elevation in plasma concentrations of the hormone. Indeed, fasting plasma ghrelin levels are higher in alcohol abstainers than in controls and ghrelin levels are positively correlated to the duration of abstinence (64, 69–71). Ghrelin elevation, however, is greater in subjects with pre-diabetes and diabetes mellitus than in normoglucose-tolerant subjects (71), suggesting that metabolic factors impact the hormone levels.

Finally, only one study to date reported that a *GHSR* gene polymorphism was more frequent in heavy drinkers than in moderate drinkers and abstinents (107), suggesting genetic association between ghrelin and heavy alcohol consumption.

In conclusion, reduced plasma ghrelin levels with alcohol consumption and elevated levels with abstinence, as well as correlation between ghrelin and alcohol craving suggest that elevated ghrelin might contribute to cravings for alcohol (Figures 2 and 3). However, plasma concentrations of DAG and obestatin, regarding alcohol status, are unknown and the contribution of these other ghrelin-derived peptides in alcohol addiction would need to be explored as well (Figure 2).

## Preproghrelin-Derived Peptides in Addiction to Drugs of Abuse

### Preproghrelin-derived peptides and consumption of drugs of abuse

#### Pharmacological actions of ghrelin in animals and humans

An emerging literature demonstrates that ghrelin modulates the action of psychostimulants such as nicotine, amphetamine, cocaine, and heroin but no data is available on the effects of the other ghrelin-derived peptides (Figure 1). In animal models, these rewarding properties of drugs of abuse are often measured by locomotor stimulation, NAcc dopamine release and/or CPP. Indeed, systemic ghrelin infusion increases cocaine-induced hyperlocomotion as well as CPP in rats [reviewed in Ref. (108)]. In addition, i.c.v infusion of ghrelin induces an increase in heroin reinforcement breakpoint on a progressive ratio schedule when animals have to work to obtain a reward (109). Bilateral micro-injection of ghrelin into the NAcc, where cocaine induces locomotor activation, increases cocaine-induced hyperactivity and these effects are blocked by a GHS-1a antagonist, [d-Lys3]-GHRP-6, in rats (110). Finally, ghrelin also amplifies nicotine-induced striatal dopamine release in this species (111).

In healthy non-smokers, after a caloric load of glucose, nicotine increases the modulatory effect of ghrelin on food-cue reactivity, measured by magnetic resonance imaging, in the ventromedial prefrontal cortex (112). No other clinical data are currently available and observations in rodents will need to be confirmed in humans.

#### Genetic or pharmacological blockade of the ghrelin/GHS-R pathway

As for alcohol consumption, GHS-R1a antagonists have been shown to suppress reward induced by cocaine and amphetamine. This was partly reviewed previously [reviewed in Ref. (91)] and recent data corroborate these findings. In rats, inactivation of ghrelin signaling by JMV2959 reduces nicotine-induced locomotor sensitization (108). In mice, the ability of nicotine, amphetamine, and cocaine to induce hyperlocomotion, to stimulate NAcc DA release and to condition a place preference is reduced after treatment with the same GHS-R1a antagonist (100, 113). In addition, in rats, the ability of morphine to induce behavioral stimulation, including stereotyped behavior and DA release in the NAcc is reduced by JMV2959 injections (114). Genetic ablation of GHS-R in mice similarly attenuates hyperlocomotion and CPP induced by nicotine, cocaine, and amphetamine (108). However, although exogenous ghrelin administration increases heroin self-reinforcement as described above, central administration of the GHS-R1a antagonist [d-Lys3]-GHRP-6, has no effect on heroin self-administration or food-deprivation induced reinstatement of heroin seeking behavior (109). Whether this lack of effect is due to the specificity of the antagonist or the nature of the psychostimulant (i.e., heroin or other) is unknown.

### Regulation of preproghrelin-derived peptides in animal and human pathophysiology

A positive correlation between plasma ghrelin levels and cocaine-seeking behavior is observed in rats trained to self-administer cocaine i.v (39).

Very little pre-clinical and clinical data are available concerning substance of abuse and ghrelin levels (Table 2). In humans, circulating total ghrelin levels are not associated with craving and withdrawal symptoms in heavy smokers suffering from acute 24 h nicotine withdrawal (72). Young adults exposed to prenatal smoke have higher plasma total ghrelin levels (73). No differences in total ghrelin levels are found between smokers and non-smokers and smoking two cigarettes acutely does not provoke any short-term changes in total ghrelin levels in smokers but induces a decline in non-smokers (74). However, nicotine administration in healthy non-smokers does not alter plasma non-acylated ghrelin levels (75). Genetic association studies only report variation of the ghrelin signaling system in individuals with amphetamine dependence (38).

## Central Mechanism of Action Mediating Reward-Relevant Behaviors

### Mesolimbic cholinergic-DA system

All major drugs of abuse acutely activate the mesolimbic dopamine system. Several lines of evidence converge to show that ghrelin activates the cholinergic –dopaminergic reward link, including a dopaminergic projection from the VTA to the NAcc to increase the consumption of rewarding foods and alcohol after i.v and i.c.v administrations in rodents (91). Ghrelin administration peripherally or locally into the LDTg concomitantly increases VTA acetylcholine as well as DA release in rats. In contrast, a GHS-R1a antagonist blocks this synchronous neurotransmitter release induced by peripheral ghrelin. In addition, local perfusion of a non-selective nicotinic antagonist mecamylamine into the VTA blocks the ability of ghrelin to increase NAcc dopamine but not VTA acetylcholine (115). The ability of alcohol to increase accumbal DA release in wild-type mice is not observed in *ghr*^−/−^ mice, suggesting that endogenous ghrelin may be required for the ability of alcohol to activate the mesolimbic DA system (116).

The mesolimbic GHS-R also plays an important role in the response to drugs of abuse. Indeed, JMV2959, a GHS-R1a antagonist, reduces morphine-induced DA release in the NAcc and behavioral stimulation, including stereotyped behavior (114). Amphetamine- and cocaine-induced locomotor stimulation and NAcc DA release, as well as the ability of these drugs to condition a place preference, are reduced in mice treated with JMV2959 in the mouse (113).

Central pathways involved in increased alcohol or other drugs consumption potentially involve several other structures and neurotransmitters, beside dopamine release in the NAcc. For example, ghrelin-induced locomotor stimulation is attenuated by VTA administration of AP5, a selective NMDA receptor antagonist, but not orexin-A antagonist or peripheral opioid receptor antagonist (naltrexone) in mice (117).

### Glutamatergic/GABAergic system

In central amygdala (CeA) GABAergic neurons, critical in regulating ethanol consumption and the response to ethanol withdrawal, ghrelin attenuates ethanol-increased IPSP amplitude and superfusion of GHS-R1a antagonists decreases IPSC and mIPSC frequency and block ghrelin-induced increases in GABAergic responses (118). Ghrelin regulation of alcohol consumption may also involve the perioculomotor urocortin neurons (pIIIu) as [d-Lys3]-GHRP-6 reduces the induction of cFos by i.p ethanol in this population of neurons but not in the VTA or arcuate nucleus of the hypothalamus (98).

### Serotonin system

The serotonin (5-HT) system is also involved in the response to ghrelin. Indeed, acute central ghrelin injections in mice increase 5-HT turnover in the amygdala and 5-HT-R mRNA in the amygdala and dorsal raphe (119). The serotonin system is also regulated by endogenous ghrelin/GHS-R signaling. Indeed in *ghsr*^−/−^ mice, decreased expression of 5-HT-R is observed in the amygdala and dorsal raphe (119).

## Preproghrelin-Derived Peptides in Stress/Anxiety/Depression

### Preproghrelin-derived peptides and stress-response/anxiety/depression

#### Pharmacological actions of ghrelin in animals and humans

Ghrelin is involved in neuroendocrine and behavioral responses to stress through activation of the HPA axis: peripheral ghrelin indeed increases hypothalamic CRH mRNA and serum corticosterone. In addition, ghrelin-induced anxiogenic effects are inhibited by a CRH receptor antagonist (120). Behavioral responses include anxiety-like behaviors like exploration in the open field, elevated plus maze (EPM), light/dark box, and social interactions.

In the mouse, both i.c.v and i.p administration of ghrelin induce anxiogenic behavior in the EPM test (120). Intracerebroventricular injections or direct injections in specific nuclei, including the hippocampus, amygdala, dorsal raphe nucleus or in several hypothalamic nuclei (Arc, PVN, VMH, PFH) induce anxiogenic responses in the open-field or EPM in both male and ovariectomized female rats (121–125), whereas i.c.v injection of obestatin elicits an anxiolytic effect in the EPM test (Figure 1) (126).

In rats, chronic i.c.v treatment with ghrelin also reveals an increase in anxiety- and depression-like behaviors that are associated with modifications in the expression of key markers involved in these behavioral changes in the amygdala (127). Interestingly, the anxiogenic actions of ghrelin are inhibited by a CRH antagonist, suggesting that anxiety response may be relayed by hyperactivity of the HPA stress axis (120). Electrophysiological responses to ghrelin challenges in the dorsal raphe, the main region expressing serotonergic neurons, which are key mediators of emotional reactivity further support a key role of ghrelin in emotional responses (127).

Despite a large literature supporting a pro-anxiety action of ghrelin in rodents, contradictory data arise from the effects of ghrelin on anxiety response (Figure 1): one study demonstrates that a subcutaneous injection of the 28-AA peptide produces anxiolytic- and antidepressant-like responses in the EPM and forced-swim test in mice (40) while in another study in food-deprived rats during 1 h between ghrelin injection and testing, a decrease in anxiety-like behavior was observed after intra-amygdala injections (122). This discrepancy needs to be clarified and may reflect differences in contextual environment during the testing period (i.e., situation where stress in present or not, see below in the next paragraph differential response in basal conditions or in response to stress).

So far, human data on the effects of ghrelin are missing and do not corroborate observations in rodents. One study demonstrates that, in male and female patients with mood depressive disorders (MDD), ghrelin administration (50 μg between 11 p.m. and 1 a.m.) induces transient GH and cortisol secretion, increases the time of sleeping in males only but has no significant effect on depressive symptoms (128). Ghrelin affects the sleep/wake pattern in healthy subjects and may have also the same effect on MDD patients but it seems independent of the etiology of the depression.

#### Genetic or pharmacological blockade of the ghrelin/GHS-R pathway

Data are currently missing concerning the effects of GHS-R1a pharmacological blockade on anxiety- and depression-like behaviors but exploration of knock-out models give some insights about endogenous ghrelin function. *ghsr*^−/−^ mice have reduced latency to leave center in the open-field test, suggesting increased anxiety in this model (129). Interestingly, a decreased expression of serotonin receptors (5 HT-R) is observed in the amygdala and dorsal raphe of *ghsr*^−/−^ mice (119). Although *ghrelin*^−/−^ mice have lower anxiety in basal unstressed conditions in three different behavioral tests (open field, EPM, light/dark box), they are also more anxious in response to acute restraint stress and show exacerbated central responses to stress as well (130). This stress response involves, not only the hypothalamus and amygdala, but also urocortin 1 neurons in the Edinger-Westphal nucleus. Interestingly, exogenous ghrelin reverses the exacerbated neuronal activation in the hypothalamic PVN and medial nucleus of the amygdala in *ghrelin*^−/−^ mice after acute restraint stress, supporting an anxiolytic action of ghrelin (130). The differential response in basal and in stress conditions suggests that the role of the ghrelin system may be different depending on the context. Ghrelin may also prevent excessive anxiety under conditions of chronic stress (see next paragraph for animal models of chronic social defeat stress). Indeed, *ghsr*^−/−^ mice display enhanced deleterious effects of chronic exposure to stress. Interestingly, increased plasma ghrelin levels induced by caloric restriction also produces anxiolytic and anti-depressant-like effects (40). Thus, stress-induced elevated ghrelin may help to control excessive anxiety and prevent depression in conditions of chronic stress exposure (Figure 3).

Finally, administration of antisense DNA for ghrelin into the lateral ventricle induces anxiolytic and antidepressant responses in the forced-swim test, EPM as well as in the black and white test and conditioned fear test in rats (131), which is in favor of an anxiogenic role of endogenous ghrelin.

### Regulation of preproghrelin-derived peptides in animal and human pathophysiology

#### Animal pathophysiology

Several studies suggest that ghrelin plays an important role in metabolic adaptations following chronic stress, which function would be to defend against depressive-like symptoms of chronic stress but which can also lead to metabolic dysfunctions in the long-term.

Animal models of anxiety/depression can be induced by chronic exposure to social stress (CSDS), a model of prolonged psychosocial stress in humans. CSDS is based on a resident intruder paradigm in which a test mouse is introduced into the cage of an aggressive CD1 mouse for a few minutes each day during several days and is a model of prolonged social defeat stress in rodents. Acylated ghrelin levels are increased in CSDS mice (40) (Table 1). Elevated ghrelin levels induced by chronic exposure to social stress are associated with increased caloric intake and body weight gain in male C57BL/6 mice and minimizes CSDS-associated depression-like behavior whereas stressed mice lacking ghrelin receptors (*ghsr*^−/−^ mice) or treated i.c.v with ghrelin receptor antagonist [d-Lys3]-GHRP-6 show attenuated weight gain and feeding responses under the same social stress paradigm (41, 132). In high-anxiety Wistar Kyoto rats, lower total ghrelin levels compared to SD low-anxiety rats in both fasted and fed states were reported (42). In rats, exposure to water avoidance stress, an acute psychological stress, mobilizes total ghrelin. Interestingly, higher plasma ghrelin levels are induced after stress in low-anxiety SD rats than in high-anxiety Wistar Kyoto rats (133), suggesting that animals with low anxiety have a greater ability to mobilize ghrelin in response to stress.

#### Human pathophysiology

##### Anxiety/stress

Although stress elevates plasma ghrelin levels in animal models, which was proposed by some studies to increase emotional eating and help defend against some depressive symptoms induced by stress, there are farther less evidence in human pathophysiology that ghrelin is involved in the eating/metabolic response to stress (Figure 2).

Psychological stress, induced by public speaking over 2 days, does not modify plasma total ghrelin levels (60). In another study performed in normal weight, obese patients and subjects with binge-eating, social stress test does not modify ghrelin levels. However, when subjects are analyzed according to their cortisol response, ghrelin levels are found increased in cortisol responders subjects following the stress whereas no change occurred in cortisol non-responders and positive correlations are found between ghrelin and cortisol change (76). In emotional eaters, evaluated by the “emotional eating subscale of the Dutch eating behaviors questionnaire,” anticipation of a psychological stressor (public speaking) leads to a greater food consumption than in-non-emotional eaters and ghrelin levels are more elevated in women anticipating the stressor compared to those not subjected to the stressor. Interestingly, the normal decline in ghrelin concentrations following food consumption is lower in non-emotional eaters (77), suggesting that ghrelin may contribute to emotional eating following a stressor (Figure 3).

##### Depression

In the majority of studies, no difference in plasma total or acylated ghrelin levels were reported between patients with major-depressive disorder (MDD) and healthy subjects (Figure 2; Table 2) (78–81). However, in MMD patients, ghrelin levels correlate positively with the severity of reduced appetite and negatively with gray matter volume of the VTA (81), and correlate with eating behavior scales like the three-factor eating questionnaire (TFEQ), suggesting that ghrelin may be associated with increased susceptibility to eating disorders observed in psychiatric patients (78).

Whether ghrelin can contribute to the degree of depression is not clearly demonstrated. In women across the weight spectrum, there was no relationship between ghrelin and symptoms of depression or anxiety (82). Interestingly, however, plasma acyl ghrelin levels were higher in treatment-resistant patients than responsive patients or in controls (83). One bias of these studies is that some of them do not discriminate ghrelin levels according to patients with or without medication (either neuroleptics, anti-depressant, antipsychotics or hypnotics). However, treatments impact plasma ghrelin levels and metabolic modifications may be secondary to the treatment. For example, in one study with MDD patients, treatment reduced plasma levels of acyl and desacyl ghrelin, as well as BMI, and ghrelin levels were lower than in controls (84). In contrast, treatment of lean patients with MDD with maprotiline, an anti-depressant, resulted in a minor increase in total ghrelin levels and WG (85). In both major-depressive episode (MDE) and bipolar disorder manic episode (BD-me), acylated ghrelin levels are decreased by electroconvulsive therapy (ECT), although BMI is unchanged (86), but remains higher than in controls.

##### Schizophrenia

Weight gain is a common side effect of the atypical antipsychotics (AAPs) used to treat schizophrenia and it has been related with the orexigenic effect of elevated serum ghrelin rather than leptin deficit (134). Among five widely used AAPs (clozapine, olanzapine, risperidone, amisulpride, or quetiapine), only the later did not elevate the ghrelin level. Ghrelin gene polymorphisms have been associated with pathogenic variations in plasma lipid concentrations, blood pressure, plasma glucose, and BMI. Four SNPs (Leu72Met, −501A/C, −604 G/A, and −1062 G > C) were genotyped in 634 schizophrenia patients and 606 control Chinese Han subjects (135). These four *GHRL* gene SNPs were not associated with SZ in this Chinese Han population. However, the −604 G/A polymorphism was associated with significant BW and BMI increases during AAP treatment.

The effect of a 16 week-treatment with olanzapine was studied by functional magnetic resonance imaging in conjunction with a task requiring visual processing of appetitive stimuli in schizophrenic patients and healthy controls (136). Neuronal activity in the fusiform gyrus was brought back to “normal” after olanzapine treatment and this change was positively correlated with the restoration in ghrelin and leptin levels, following the treatment. However, others have reported that serum leptin levels might be a more sensitive biomarker than ghrelin or adiponectin levels to differentiate schizophrenic patients and healthy controls (137).

In some of the cited disorders, we need to be precautious with the interpretation because they concern small groups of subjects and inter-individual variability in the ghrelin pattern has to be considered (Table 2).

## Ghrelin-GH Axis and Psychiatric Disorders

As ghrelin has initially been described as a powerful GH secretagogue and a number of studies have focused on its role as a regulator of the GH/IGF-1 axis (1, 13, 138), the link between ghrelin, GH axis, and psychiatric disorders needs to be questioned. The GH/IGF-1 axis is deregulated in anorexia nervosa and the evolution of GH levels during renutrition is predictive of short-term outcome in AN-R patients (139, 140). More specifically, low GH levels at admission and absence of GH reduction after weight recovery could be predictive of short-term relapse. In patients with depression or post-traumatic stress disorder (PTSD), sleep-related GH secretion is lower compared to healthy controls (141). Abnormal IGF-1 levels have also been reported in several psychiatric disorders like schizophrenia and major depression (142). Interestingly, in GH-deficient patients symptoms of depression and cognitive impairments are improved after GH therapy (143).

Several components of the GH/IGF-1 axis also modulate anxiety and depression. At the hypothalamic level, GH secretion is directly regulated by two antagonistic neurohormones: GHRH stimulates whereas somatostatin inhibits GH secretion. Intracerebroventricular and intra-amygdala administrations of somatostatin induces anxiolytic and anti-depressant effects in rats (144, 145) while somatostatin KO mice show hyperactivity and anxiety-like behavior (146). GHRH KO mice display anxiety and depression-related behaviors (147). In addition, the GHRH agonist, JI-34, induces anxiety and depression (148) whereas the GHRH antagonist, MZ-4-71, elicits anxiolytic and anti-depressant effects (149).

Interestingly, deficiency in circulating and hippocampal IGF-1 induced by virus-mediated IGF-1 KO is associated with depressive symptoms measured in the forced-swim test in mice (150). A recent study brings an interesting point of view on the link between ghrelin–GH axis and stress-associated mental diseases. In a rodent model of PTSD, in which rats are repeatedly exposed to a stressor and display enhanced fear, stress-related increases in circulating ghrelin are necessary and sufficient for stress-associated vulnerability to exacerbated fear learning. These actions of ghrelin require GH in the amygdala to exert fear-enhancing effects, suggesting that ghrelin–GH axis can mediate maladaptive changes following prolonged stress (151).

## Conclusion

Ghrelin interacts with the GHS-R to modulate GH secretion, natural and artificial reward as well as stress and anxiety. Anhedonic symptoms, which include loss of pleasure, appetite, and motivation, are often observed in these disorders in association with altered ghrelin secretion and/or signaling. Whether dysfunction of the ghrelin/GHS-R signal contributes to the mechanism responsible for the development of the disease or can help to minimize some symptoms associated with these psychiatric disorders is still debated.

The recent demonstration of the heterodimerization of the GHS-R and the dopamine D2-receptor requested for appetite regulation in animal is a novel avenue for future studies deciphering the role of ghrelin in reward and addictions that are both impaired in psychiatric disorders. The multiple links between the ghrelin/GHS-R system and other biological pathways, via the functional heterodimerization with other receptors that could play a role in psychiatric disorders, would be interesting to explore.

Finally, although ghrelin has been proposed to be a pharmacological target for treatment of psychiatric disorders, only a few data on the involvement of the other ghrelin-derived peptides, DAG, and obestatin, in psychiatric disorders are available. This would require further investigations.

## Conflict of Interest Statement

The authors declare that the research was conducted in the absence of any commercial or financial relationships that could be construed as a potential conflict of interest.
